# Anatomy of the Macular Ganglion Cell Layer Plexus on Projection-Resolved Optical Coherence Tomographic Angiography

**DOI:** 10.1167/tvst.14.6.5

**Published:** 2025-06-02

**Authors:** Jie Wang, Karine D. Bojikian, Aiyin Chen, Ping Wei, Liang Liu, Yali Jia, David Huang

**Affiliations:** 1 Casey Eye Institute, Oregon Health & Science University, Portland, OR, USA; 2 Department of Biomedical Engineering, Oregon Health & Science University, Portland, OR, USA

**Keywords:** ganglion cell layer plexus (GCLP), projection-resolved optical coherence tomographic angiography (PR-OCTA), vessel density (VD)

## Abstract

**Purpose:**

The purpose of this study was to characterize the central macular ganglion cell layer plexus (GCLP) boundaries using projection-resolved optical coherence tomographic angiography (PR-OCTA) in healthy eyes.

**Methods:**

Participants were scanned using a commercial OCTA system (Avanti; Optovue/Visionix Inc., Fremont, CA, USA) in a 6 × 6-mm area centered on foveal fixation.PR-OCTA algorithm was used to remove flow projection artifacts. The anterior GCLP boundary was marked at the nerve fiber layer (NFL)/ganglion cell layer (GCL) junction, and the posterior boundary with the intermediate capillaries plexuses (ICPs) was determined by searching the ganglion cell and inner plexiform layer (GCIPL) slab for the watershed depth with minimum vessel density (VD). The foveal avascular zone (FAZ) within a 1-mm diameter circle was excluded from the analysis because the retinal plexuses merge near the FAZ. A polynomial fit was used to model the relationship between VD and depth.

**Results:**

Thirty-eight eyes of 38 healthy participants (79% female subjects) were enrolled. The mean age and standard deviation were 59.6 ± 10.7 years. The watershed between the GCLP and ICP was located at 75% depth within the GCIPL throughout the macula. Analysis of macular sectors on a polar grid showed that GCLP VD was correlated with NFL, GCIPL, and macular ganglion cell complex (GCC) thicknesses (*R*^2^ = 0.26, 0.15, and 0.73, respectively, *P* < 0.01 for all). The correlation was significantly stronger for GCC.

**Conclusions:**

We defined the anatomic GCLP slab in the macula on PR-OCTA, which is the anterior 75% of the combined GCIPL. Its density correlates best with GCC, which also contains the NFL, suggesting that it also supplies at least the posterior aspect of the NFL.

**Translational Relevance:**

Mapping macular GCLP holds promise for evaluating perfusion, particularly in conditions such as glaucoma and optic neuropathies.

## Introduction

Retinal ganglion cells (RGCs) are the neurons that relay information from the retina to the brain.^1^^,^^2^ Their bodies reside in the ganglion cell layer (GCL),^3^^–^^5^ the axons coalesce in the nerve fiber layer (NFL),^6^^,^^7^ and the dendrites synapse with bipolar cells and amacrine cells in the inner plexiform layer (IPL). Together, the IPL, GCL, and NFL are called the ganglion cell complex (GCC). The macula is the central region of the retina and one of the most metabolically active tissues in the human body. It contains more than 30% of the RGCs.^8^^–^^10^ Optical coherence tomography (OCT)^11^ and OCT angiography (OCTA)^12^ are noninvasive 3-dimensional imaging modalities that are used to evaluate the structure and perfusion of the macular GCC and peripapillary NFL, which are damaged in glaucoma and other optic nerve diseases.^13^^,^^14^

The flow projection artifact limits the 3-dimensional visualization of vascular network with OCTA.^15^^,^^16^ The flow signal in OCTA arises from the variation in the amplitude and phase of backscattering of moving blood cells. This signal is propagated to distal structures because moving blood cells also cause variable forward scattering and shadowing. Fortunately, this flow projection artifact can be greatly reduced with special software that resolves the in situ flow signal from the projected flow signal.^17^^,^^18^

Our previous studies using projection-resolved OCTA (PR-OCTA) had demonstrated four distinct vascular plexuses in the inner retina, including the NFL plexus (NFLP), GCL plexus (GCLP, previously referred to as the superficial vascular plexus or SVP), intermediate capillary plexus (ICP), and deep capillary plexus (DCP).^17^^–^^20^ The NFLP and GCLP together make up the superficial vascular complex (SVC), which perfuses the GCC. In our previous work, we had empirically established that the SVC occupies approximately the anterior 80% of GCC.^21^ The posterior boundary of SVC and the ICP was established by locating the depth of the watershed (depth of least vessel density) at sampled locations in the macula.

In this study, we aim to further define the structure and function of GCLP in the macula using PR-OCTA. It is desirable to separately measure the NFLP and GCLP because they primarily perfuse separate retinal layers with distinctly different functional distributions. Nerve fiber bundles in the NFL radiate in arcuate trajectories over wide retinal regions. Therefore, NFLP measurements provide good global or regional assessments. In contrast, RGCs in the GCL connect to the local retinal circuitry. Therefore, the GCLP would be more tightly linked to local retinal function than the NFLP. We already know that the junction of the GCLP and NFLP occurs at the GCL-NFL boundary. There is no watershed at this boundary, but the organization of the capillaries abruptly changes between the NFLP (parallel to nerve fibers) and the GCLP (nondirectional). The posterior boundary of the GCLP is in the IPL, but the depth has not been precisely defined and is a topic of investigation in this paper.

## Methods

### Data Acquisition and Pre-Processing

Healthy eyes were studied, one from each healthy subject. The inclusion criteria included normal intraocular pressure, normal corrected distance visual acuity, and normal visual field (Humphrey Field Analyzer II, SITA 24-2 test). The study was conducted in compliance with the Declaration of Helsinki.

Participants were scanned using the 70 kilohertz (kHz) commercial spectral-domain OCTA system (Avanti, Optovue/Visionix Inc.) with a central wavelength of 840 nm. Eyes were scanned using the 6 × 6-mm high-definition (HD) macular OCTA scan pattern that was centered on the foveal fixation point and consisted of 400 A-lines in each B-scan and 400 B-scan locations in each volumetric scan. In the OCTA scans, two B-scans were acquired at each location, and the commercial split-spectrum amplitude-decorrelation angiography (SSADA) algorithm was applied to calculate the flow signal by analyzing variations between the two B-scans.^12^ One X-Fast and one Y-Fast volumes were acquired, registered, and merged to remove motion artifacts.^22^ Two repeats were acquired from each subject for measuring the repeatability.

Three retinal layer boundaries, including inner limiting membrane (ILM), NFL/GCL, IPL/inner nuclear layer (INL), were automatically segmented using our custom OCTA reading software toolkit.^23^ Segmentation was reviewed by a certified human grader and found to be completely accurate. Therefore, no manual correction was applied.

**Figure 1.**
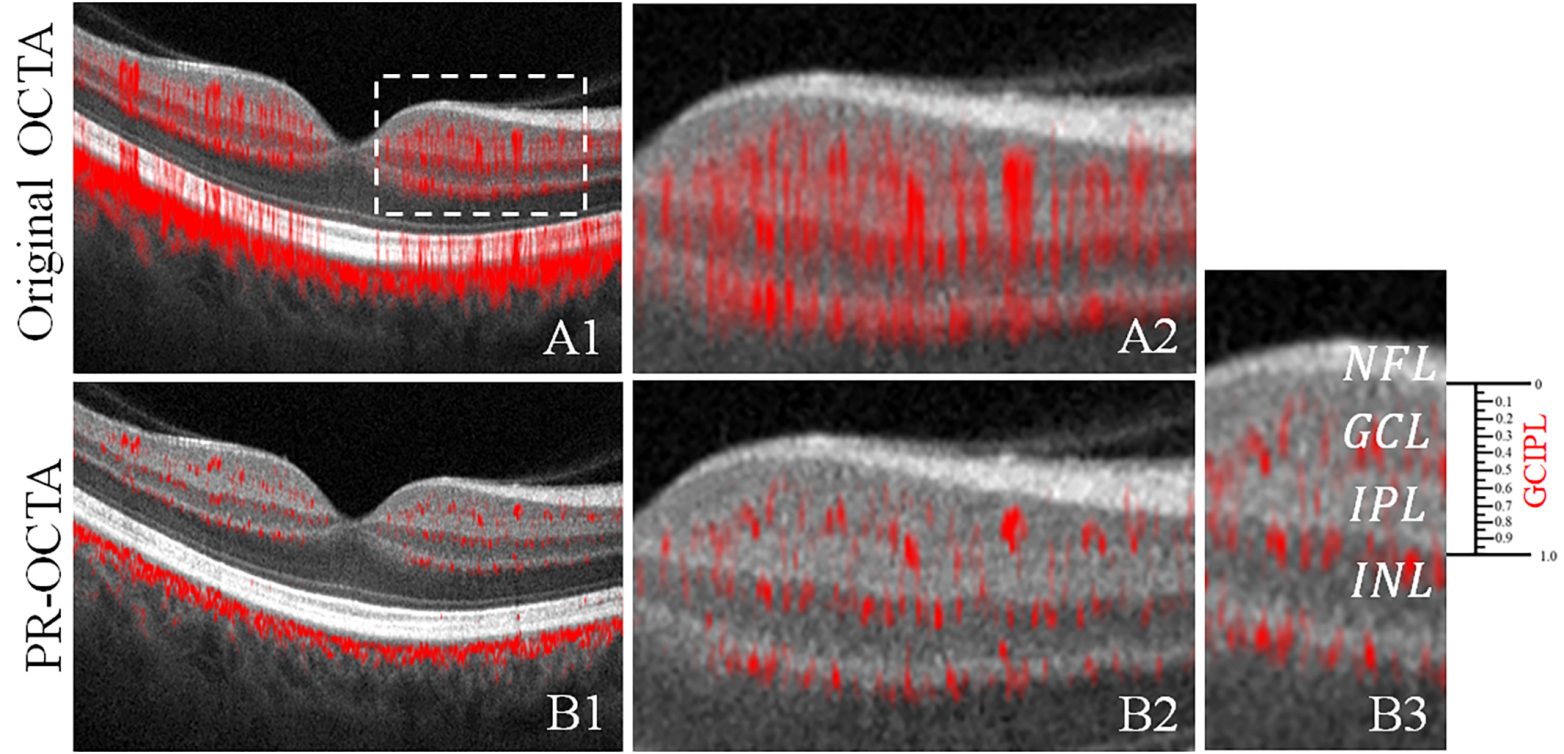
Demonstration of the original OCTA and projection-resolved OCTA (PR-OCTA). (**A1**) Cross-sectional structural OCT overlaid with the original OCTA signal (*red*). (**A2**) Magnified region on **A1** to show the original OCTA signal in inner retina (*outline by the white rectangle*). (**B**) Cross-sectional structural OCT overlaid with the PR-OCTA signal. (**B2**) Magnified region on **A1** to show the PR-OCTA signal in the inner retina (*outline by the white rectangle*). (**B3**) The GCIPL division in depth. GCL, ganglion cell layer; INL, inner nuclear layer; IPL, inner plexiform layer; NFL, nerve fiber layer; OPL, outer plexiform layer. The GCILP slab was divided into 20 sub-slabs for quantifying the vessel density in depth.

Projection artifacts, originally presented as “tails” in the inner retina (Fig. 1A), were removed using the PR-OCTA algorithm, which enabled clean visualization of the vasculature in the deeper plexuses below the NFLP (Fig. 1B).

### Anatomic Localization of the Ganglion Cell Layer Plexus

The anterior boundary of the GCLP is defined as the boundary between the GCL and the NFL. Because the NFL reflectance is much higher than the GCL, the boundary is easily detected on structural OCT by the image processing software. However, the reflectance of GCL is only slightly lower than the IPL, making segmentation less reliable. Therefore, we choose to define the posterior boundary of the GCLP in terms of depth within the combined ganglion cell and inner plexiform layer (GCIPL). The posterior boundary of this anatomic layer can be reliably detected because the reflectance of the IPL is much higher than that of the INL.

**Figure 2.**
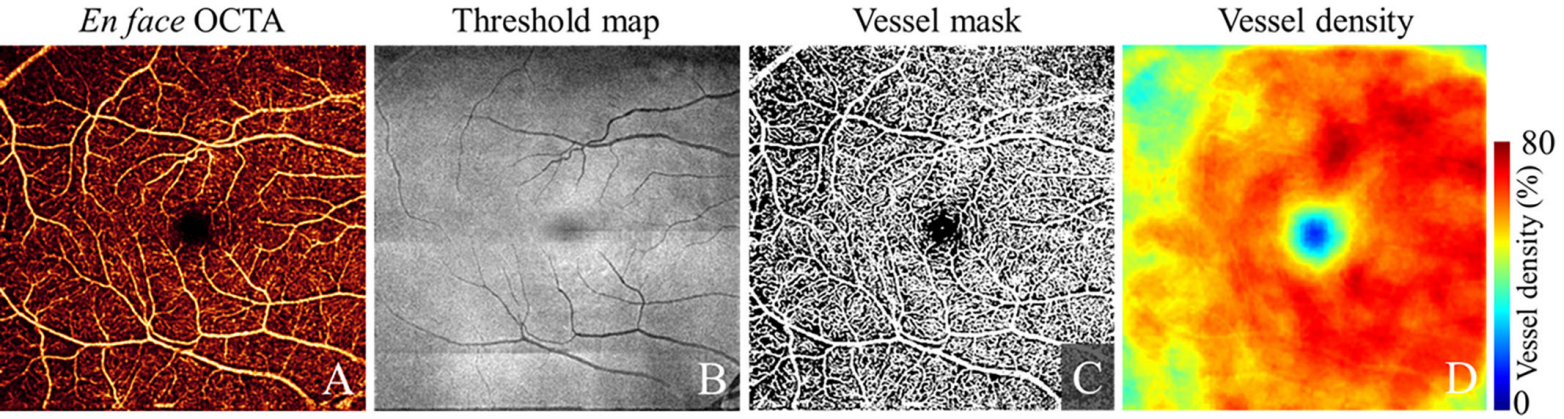
Reflectance-compensated vessel density quantification on the 6 × 6-mm macular OCTA scan in an example eye. (**A**) En face projection-resolved OCT angiogram. (**B**) Reflectance-adjusted threshold map was estimated according to the mean projection of OCT signal in a slab that includes the inner nuclear layer (INL) and photoreceptor inner segment and layers in between. (**C**) Binary vessel mask. and (**D**) Vessel density (VD) map. The VD represents the percentage or fraction of area on the en face angiogram that is occupied by vascular pixels.

We defined the watershed depth that separates the GCLP and ICP by searching for the depth within the GCIPL where the vessel density (VD) reaches a minimum value. The VD is defined as the fraction or percentage of the area on an en face OCT angiogram that is occupied by vascular pixels. To generate the VD depth profiles, the GCIPL slab was equally divided into 20 sub-slabs (Fig. 1C). An en face OCT angiogram of each sub-slab was generated using maximum flow signal projection of the projection-resolved OCTA signal (Fig. 2A). A binary vessel mask was generated from the angiogram using an adaptive threshold map that compensates for OCT signal strength variation by analyzing retinal reflectance (Figs. 2B, 2C).^24^ An averaging low pass filter was applied to the vessel mask to generate the vessel density map (Fig. 2D). Areas of very low OCT signal that do not allow accurate vessel detection (e.g. shadowing due to vitreous floaters) were detected and excluded from further data analysis.^25^

**Figure 3.**
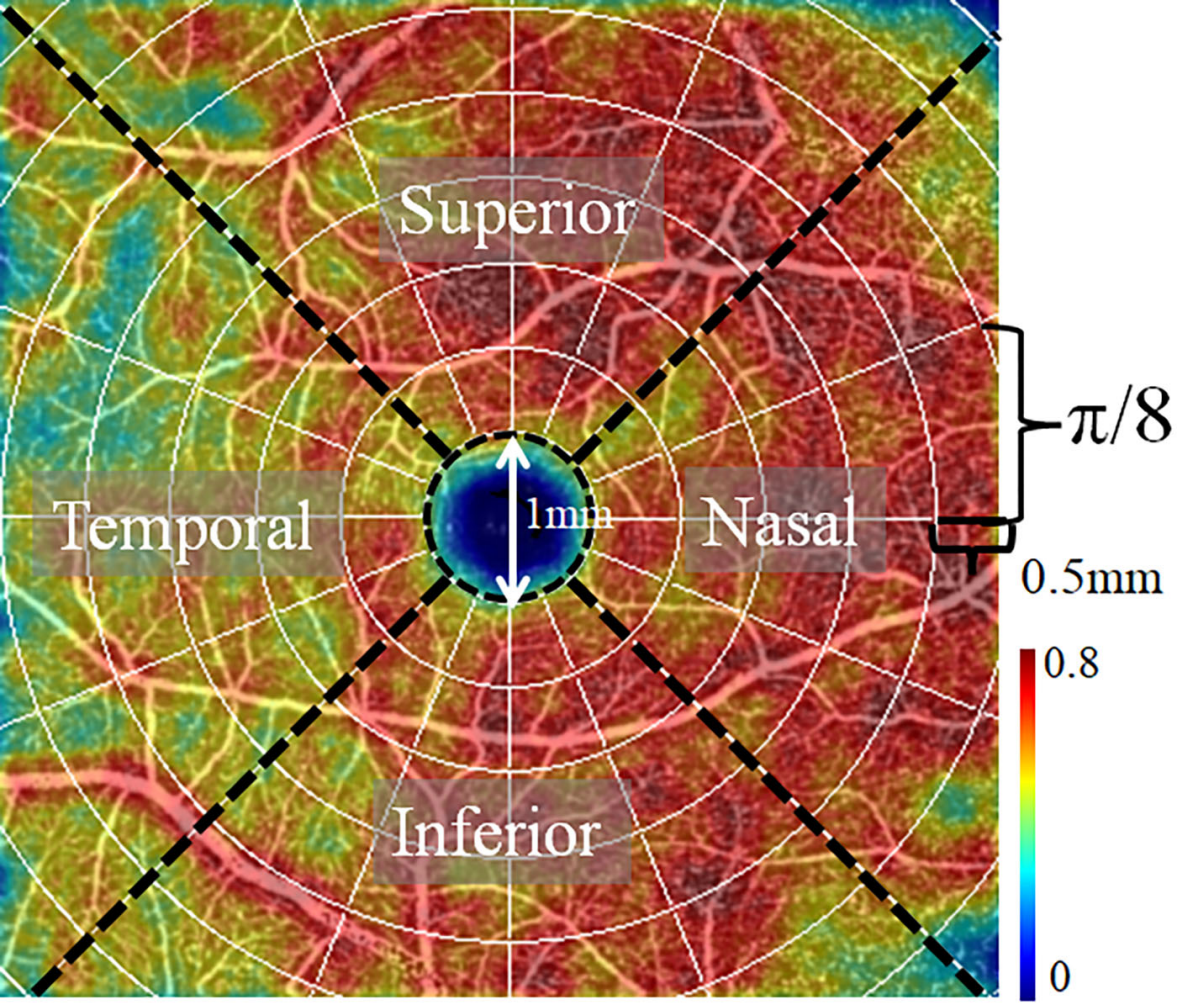
Polar sectors were used to analyze the vessel density (VD) map on a 6 × 6-mm macular scan in an exemplar eye. The color-coded VD map was overlaid on the en face OCT angiogram. The polar sectors were generated with radial annuli of 0.5 mm increments and π/8 increments of angular slices. The *circle area* with 1-mm diameter centered on the FAZ was excluded to avoid the FAZ. The VD map was generated in the whole GCIPL slab.

To analyze the VD variation in depth, VD maps of each eye were divided into polar sectors centered on the foveal avascular zone (FAZ).^26^ The sector division was based on radial increments of 0.5 mm and angular division of the circle into 16 slices (Fig. 3). The central 1-mm diameter circle was excluded because the retinal plexuses merge near the FAZ.^27^ The average VD in each sector was used in the analysis to minimize the pixel-level noise-induced variation and ensure reliable results.

The VD as a function of depth within the GCIPL slab, for each sector, was fitted with a sixth degree polynomial model by least-square regression. The depth of the watershed between the GCLP and the ICP is then identified as the minimum point on the polynomial curve.

## Results

Thirty-eight participants (79% female subject) were enrolled with ages of 59.6 ± 10.7 years (mean ± standard deviation). Thirty-eight eyes were studied and they had the following characteristics: 71% right eyes, intraocular pressure = 14.6 ± 2.17 millimeters of mercury (mm Hg), axial length = 23.7 ± 1.1 mm, spherical equivalent refractive error of −0.58 ± 1.62 diopters (D), and corrected distance visual acuity = 84.1 ± 4.6 (Early Treatment Diabetic Retinopathy Study [ETDRS] letter scores).

**Figure 4.**
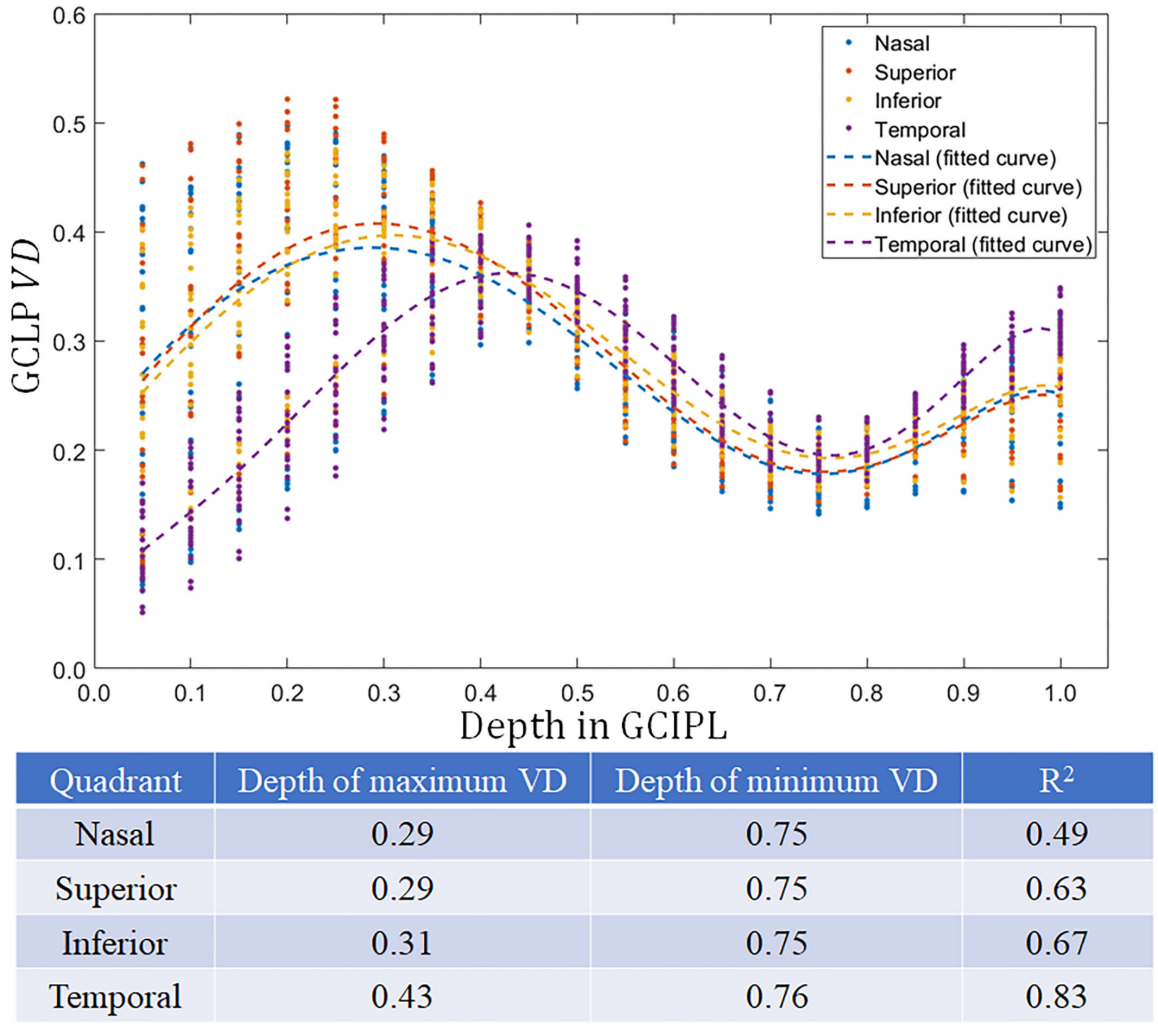
Scatterplot of vessel density (VD) as a function of depth with each point representing a polar-sector macular location averaged over all study participants. A sixth-degree polynomial was used to model the relationship between VD and depth in the ganglion cell inner plexiform layer (GCIPL) slab in each quadrant (*P* values are < 0.01).

The depths of local maximum and minimum VD within the GCIPL are shown in Figure 4. The local maximum VD depth was 33.0 ± 6.7% and noticeably deeper in the temporal quadrant. The local minimum VD depth was 75.3 ± 0.5%.

**Figure 5.**
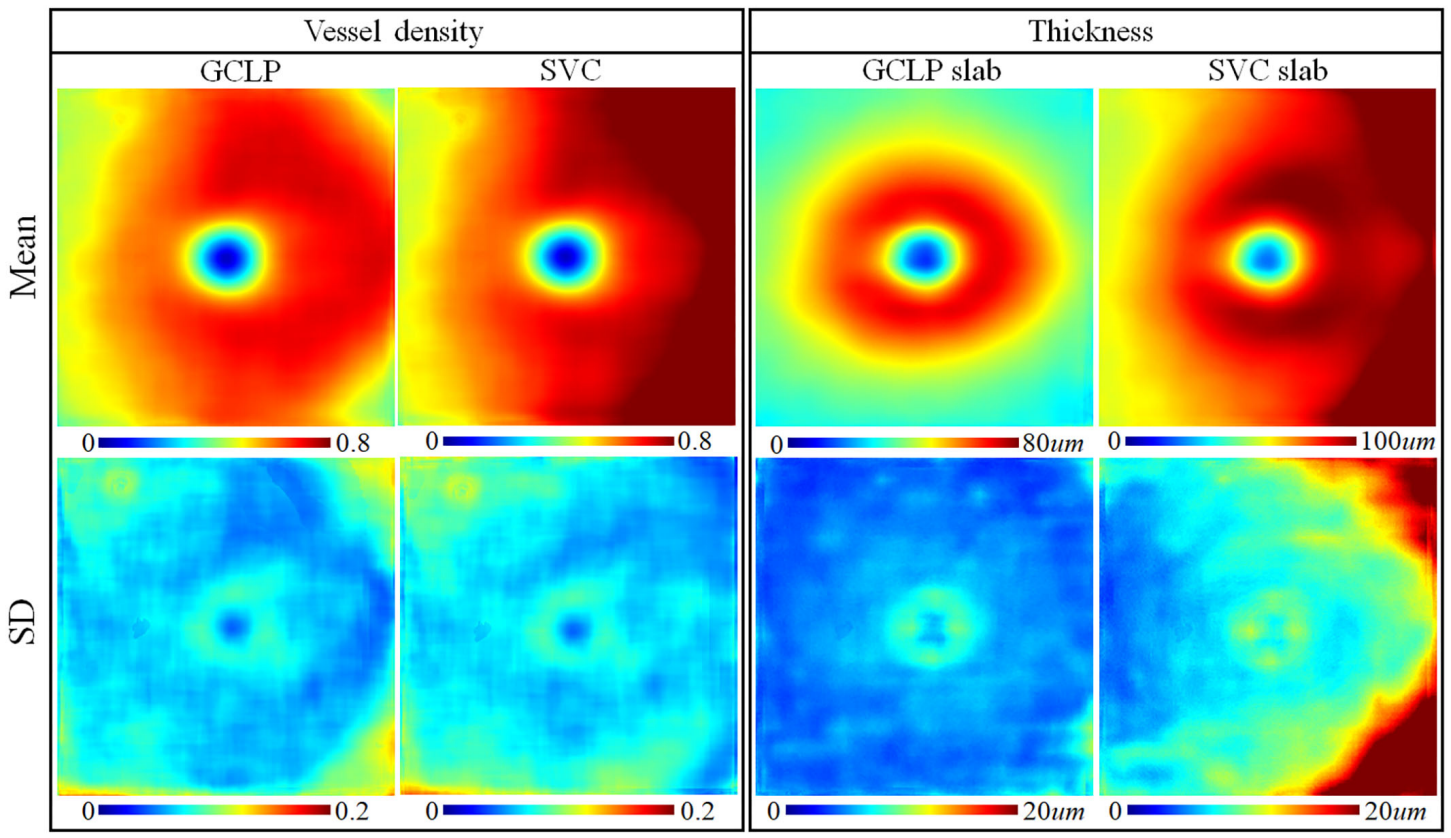
Vessel density and thickness maps of the ganglion cell layer plexus (GCLP) and superficial vascular complex (SVC) averaged over all study eyes. Population standard deviation (SD) maps are also shown to reflect the variability among study participants. Left eyes were flipped to mirror the right eyes.

The VD depth profile, averaged over study participants, is plotted in Figure 5. Polynomial curves were fit by quadrant. Peak VD in the GCLP was located at 29% to 31% of the GCIPL depth in the nasal, superior, and inferior quadrants. It was deeper (43% GCIPL depth) in the temporal quadrant, and the *P* value was < 0.01 compared with the other 3 quadrants. The local minimum VD was located at 75% to 76% depth in all quadrants, marking the watershed boundary between the GCLP and the ICP. From the nadir at the GCLP/ICP watershed boundary, the VD increased with depth toward the IPL/INL anatomic boundary. The polynomial fit shows a high coefficient of determination (*R*^2^) between 0.49 and 0.83. Based on these results, we define the boundary between the GCLP and the ICP at 75% depth within the GCIPL slab.

**Figure 6.**
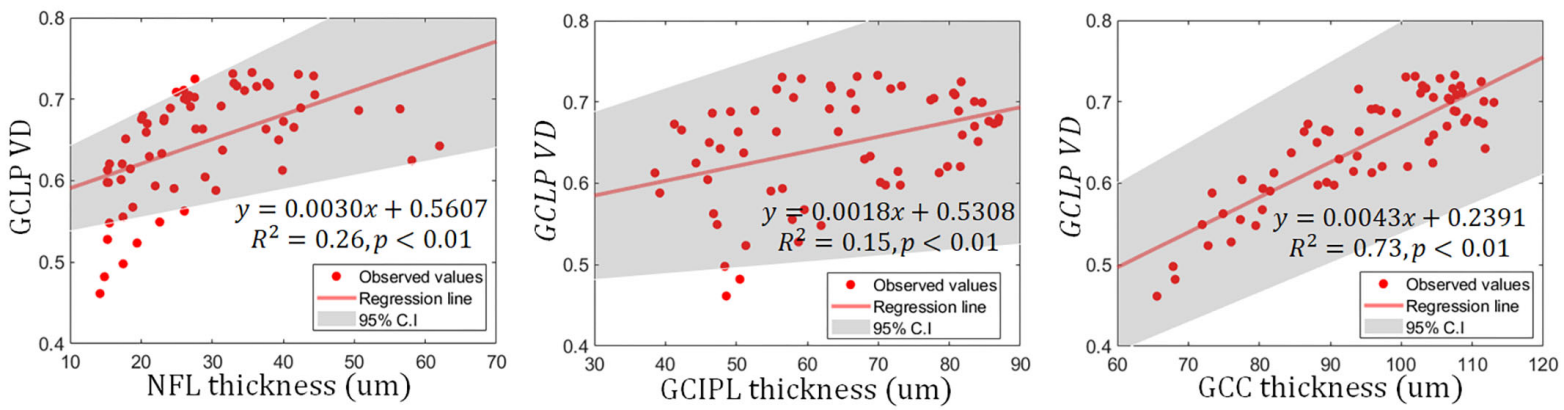
Sector-wise scatterplots of the ganglion cell layer plexus (GCLP) vessel density and nerve fiber layer (NFL) thickness, the ganglion cell-inner plexiform layer (GCIPL) thickness, and the ganglion cell complex (GCC) thickness. Each point represents the average over all participants for that sector.

Applying the newly defined GCLP slab, we now investigate its thickness and VD distribution in the macula (Fig. 6). The SVC, which includes both the GCLP and the NFLP, is also shown for comparison. The GCLP slab is thickest in the parafoveal annulus. The SVC slab shows the same annular pattern added to thick wedges on the nasal side that corresponds to the superior and inferior arcuate nerve fiber radiations. The SVC VD pattern is nearly the same as the thickness pattern. The GCLP VD is highest in the parafoveal annulus, but also shows higher density on the nasal side, where the NFL is thicker. The GCLP VD is 10% higher (*P* < 0.01) on the nasal side than on the temporal side. The population standard deviation (SD) maps of both the GCLP and the SVC show slightly higher variability in the corners. The overall root-mean-squared population variability of the GCLP VD (7.4%) and the SVC VD (7.6%) maps was similar.

We next examine whether PR-OCTA was needed to measure the GCLP (whether the project artifacts from the NFLP were significant). Without projection resolution, the GCLP VD was 68.3 ± 4.2%, significantly (*P* < 0.01, paired *t*-test) higher than the GCLP VD of 61.7 ± 3.4% measured using the PR-OCTA algorithm. For comparison, the SVC VD was 70.4 ± 4.5% (which was the same with or without projection resolution).

The within-visit repeatability for the measurements of the SVC VD, GCLP VD, GCIPL thickness, and GCC thickness were all good (see the Table). The repeatability of GCLP VD was significantly (*P* < 0.01) worse than the SVC VD based on coefficient of variation (CV). We examined the relationship between the GCLP VD and the thickness of retinal layers at all sector locations (see Fig. 5). Compared with its correlations with the GCIPL and NFL thickness (see Fig. 6), the GCLP VD is significantly (*P* < 0.01) better correlated with the GCC thickness (Pearson *R*^2^ = 0.79).

**Table.** Repeatability of Vessel Density and Thickness Measurements

	SVC VD	GCLP VD	GCIPL Thickness	GCC Thickness
CV	0.05	0.09	0.04	0.03

CV, Coefficient of variation; GCC, ganglion cell complex; GCLP, ganglion cell layer plexus; GCIPL, ganglion cell and inner plexiform layer; SVC, superficial vascular complex; VD, vessel density.

## Discussion and Conclusions

**Figure 7.**
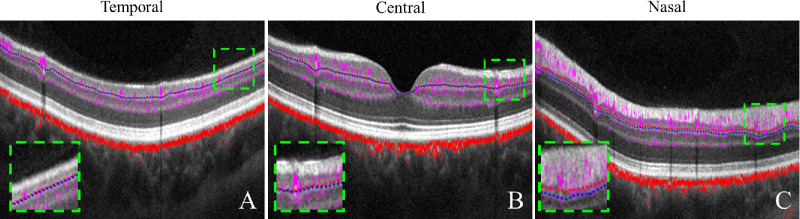
Comparison of the outer boundaries of 80% GCC (*red dotted line*) and 75% GCIPL (*blue dotted line*) on the cross-sections across the temporal (**A**), central (**B**), and nasal (**C**) macula. The definition of 80% GCC is too shallow in the nasal region where the NFL is thick, while showing subtle differences when applied to the temporal and central cross-sections. The *green rectangle regions* are magnified at the *bottom left* to highlight these differences.

A major goal of this study was to determine the anatomic landmark that could be used to demarcate the posterior boundary of the GCLP with the ICP. This was tricky because the plexuses do not correspond strictly to anatomic layers. The ICP straddles the IPL and INL, and the GCLP resides in both the GCL and IPL. As a result, the GCLP-ICP boundary is located within the IPL, a layer that is not easy to separate reliably from the GCL by automated volumetric segmentation at the resolution and sampling density of standard clinical OCT. Fortunately, the GCLP-ICP boundary is located at approximately 75% depth in the combined GCIPL, an anatomic slab that can be reliably segmented in standard scans. This depth is remarkably consistent throughout the macula, making it relatively simple to accurately segment the GCLP slab, the first step toward accurate measurement of perfusion parameters such as vessel density. Within the 6 × 6-mm macula imaging area, this new definition of the posterior of the GCLP boundary of 75% of the GCIPL depth is more precise and supersedes our previous definition of 80% GCC depth,^21^ which is too shallow when applied to regions where the NFL is thick (superior and inferior arcuate bundle regions, Fig. 7C), while showing subtle differences when applied to the temporal macula (Fig. 7A).

We revised the outer boundary of the GCLP (also known as [a.k.a.] the SVP) to 75% of the GCIPL depth rather 80% of the GCC depth. This change results in minimal differences in VD maps, resulting in slight differences in the nasal area near the optic disc, where the NFL is thicker than the GCIPL and therefore 75% of the GCIPL is posterior to 80% of GCC and results in slightly thicker GCLP slabs and higher VD. The purpose of this paper is to provide a comprehensive and precise characterization of the GCLP, which is worth putting efforts into improving previous definitions, as OCTA is still a relatively new technology just at the beginning of clinical adoption.

It is important to note that the measurement of GCLP VD requires the removal of projection artifacts arising from the NFL using the PR-OCTA algorithm (also called projection artifact removal or PAR on some OCTA systems). Without this step, projection artifacts from the NFLP would cause an over-estimation of the GCLP VD. In contrast, the measurement of 2-dimensional SVC VD (% area) does not require projection resolution because the overlap between the NFLP and the GCLP blood vessels does not affect the enumeration of vascular pixels in the combined en face slab projection. However, if we were to measure the 3-dimensional SVC VD (% volume), the projection resolution would again be required for accurate counting of vascular voxels.

The GCLP VD should be a better measure of local RGC function than the SVC because it excludes the NFLP that supplies wide-ranging retinal nerve fibers. However, we found evidence that the GCLP not only supplies the local RGC, but also partially the overlying nerve fibers. First, although the GCLP VD is highest in the annular parafoveal region where GCIPL is thickest and RGC concentration is highest, it is also higher in the nasal macula where the NFL is thicker than in the temporal macula. Second, the GCLP VD is significantly higher in the nasal half of the macula, where the NFL is thicker. Third, correlation analysis showed that the GCLP VD is better correlated with the SVC thickness rather than the GCLP thickness. Fourth, the peak GCLP VD is more posterior in the temporal quadrant, where the NFL is thinnest. The linkage between the GCLP and the NFL is not surprising in view of our previous investigation of the NFLP.^26^ We found that the NFLP is absent in areas where the NFL is less than 18 µm in thickness. Because the NFL lacks capillaries in these areas, the metabolic needs must be supplied by the GCLP. Taking into account all of this information, we believe there is strong evidence that the GCLP VD is linked to the metabolic demands of the RGC, the anterior aspect of the IPL, and the posterior aspect of the NFL.

To serve as a clinical measure of RGC perfusion, the GCLP VD measurements must have good repeatability as well as a reasonably narrow distribution within the healthy population. The within-visit repeatability for the GCLP VD measurement was good, but not as good as the SVC VD. So, there may be an advantage in using the SVC VD to track disease progression. The population variability of the GCLP VD map is not uniform within the 6 × 6-mm map, but is greatest at the corners and in the fovea. This may be due to interindividual variation in nerve fiber distribution and foveal circulation. In some subjects, a reduced OCT signal level in the corners of the macular scan due to pupil edge vignetting may also play a role. To construct optimal diagnostic parameters, these areas of higher variability may be excluded from analysis.

The GCL has the greatest capillary density of all retina layers in the human retina,^28^ and the need of high capillary density translates in the RGCs being vulnerable to mild hypoxic stress.^29^ Better understanding the macular RGCs’ blood supply has implications in diseases, such as glaucoma and other optic neuropathies. Previous OCT studies in patients with glaucoma had shown thinning of the GCC and GCIPL.^30^^–^^32^ Being able to measure the perfusion in the specific layers where the RGCs reside has the potential for detecting the reduced metabolic rate in dysfunctional RGCs even before they undergo apoptosis. Previous OCTA studies in patients with glaucoma and patients with compressive optic neuropathy have reduced VD in peripapillary NFL and macular GCC,^33^^–^^37^ sometimes preceding the anatomic changes.^38^ Improvement in GCLP VD in response to treatment could reflect reversal of RGC dysfunction. Such improvements have been shown in the analysis of peripapillary NFL VD.^33^^,^^34^^,^^39^^,^^40^ Mapping of macular GCLP VD has the potential advantage of being better correlated with local RGC dysfunction and visual field defect than mapping of the peripapillary NFLP and the macular SVC.

To the best of our knowledge, we are the first group to report anatomic retinal circulation in cross-section in vivo using PR-OCTA.^21^ We initially defined the retinal vascular network in four plexuses: the peripapillary capillary plexus (RPCP),^26^ the SVP, the ICP, and the DCP. But we have since renamed the plexuses to be more consistent with their anatomic distribution.^20^^,^^41^ We renamed the RPCP the NFLP because the bulk of this plexus radiates beyond the peripapillary region and serves the arcuate nerve fiber bundles where the NFL is thick.^26^ Because the SVP is deeper than the NFLP, it is inappropriate to characterize it as “superficial.” Thus, we renamed the SVP as the GCLP.^20^^,^^41^ Together, the NFLP and the GCLP constitute the SVC, which perfuses the retinal ganglion cells within the GCC comprising the NFL, GCL, and IPL.

It is useful to separately measure the VD of the NFLP and the GCLP because they relate differently to the retina and the visual field. The NFLP supplies the wide-ranging nerve fibers that originate from ganglion cells all over the retina and converges on the optic nerve head and the peripapillary NFL. In contrast, the GCLP primarily supplies the local ganglion cells and is therefore tied to the nearby visual field. Our previous studies have shown that analyzing the peripapillary NFLP loss can provide accurate glaucoma diagnosis and is useful in simulating the wider 24-2 visual field.^34^^,^^42^ More recently, we have also demonstrated sensitive detection of focal damage and accurate glaucoma diagnosis by analyzing the NFLP vessel density map.^43^ We anticipate that macular NFLP analysis would be useful for simulating the central 10-2 visual field. Given the potential of GCLP as a new biomarker for assessing glaucoma and other optic neuropathies, fundamental investigations to more accurately define its boundary and characteristics are needed.

Our study has some limitations. Our sample size was relatively small and a larger study population is needed to establish a normative database of the GCLP VD and the effects of demographic variables (i.e. age, gender, and race) and anatomic covariates (e.g. axial length and optic disc size). Nevertheless, the basic characterization of the GCLP set forth in this paper introduces a new tool for future investigations in glaucoma and other optic nerve diseases.
